# Advances in Nutrition for Metabolic Dysfunction-Associated Steatotic Liver Disease (MASLD) Management: Current Perspectives from the Bench to the Bedside

**DOI:** 10.3390/nu18111708

**Published:** 2026-05-27

**Authors:** Ildefonso Rodriguez-Ramiro

**Affiliations:** Department of Nutrition and Food Science, Faculty of Pharmacy, Complutense University of Madrid, 28040 Madrid, Spain; ildeforo@ucm.es

## 1. Introduction

Metabolic dysfunction-associated steatotic liver disease (MASLD), formerly known as non-alcoholic fatty liver disease (NAFLD), has emerged as the most prevalent chronic liver disease worldwide, with an overall global prevalence of 30% [1,2]. It encompasses from hepatic steatosis, through to metabolic dysfunction-associated steatohepatitis (MASH), the progressive necroinflammatory disease form that can progress to fibrosis, cirrhosis, and hepatocellular carcinoma [1]. It is recognized as a complex systemic metabolic disorder closely linked to obesity, insulin resistance, cardiometabolic dysfunction, gut microbial dysbiosis, and genetic risk factors [1]. Despite recent advances in pharmacological therapies, including the approval of agents such as “resmetirom”—the first approved therapy for noncirrhotic MASH with moderate-to-advanced fibrosis [3]—lifestyle modification remains essential for MASLD prevention and treatment [4]. Within this lifestyle modification framework, nutrition plays a central and multifaceted role, acting on key pathophysiological mechanisms including hepatic lipid metabolism, oxidative stress, inflammation, mitochondrial dysfunction, and gut–liver axis regulation. The present Special Issue of *Nutrients*, entitled *Advances in Nutrition for MASLD Management: Current Perspectives from the Bench to the Bedside*, brings together six contributions that collectively provide a comprehensive overview of nutritional strategies across different levels of biological and clinical complexity and from pre-clinical murine studies towards a scoping review of primary trials.

## 2. Mapping the Landscape of Bioactive Compounds

The expanding interest in dietary bioactive compounds is comprehensively captured by the systematic scoping review by Handu et al. (Contribution 1). This review examined the current evidence on bioactive-substance-based interventions in MASLD. By synthesizing over 180 studies, including primary trials (randomized controlled trials (RCTs) and controlled trials) and systematic reviews, the authors highlighted extensive research on compounds such as curcumin, silymarin, and resveratrol. The review systematically summarized the outcomes reported for bioactive compounds supported by at least three published studies in MASLD management. While these clinical investigations provide evidence of beneficial effects on hepatic and metabolic outcomes, many of the molecular mechanisms proposed to underline these effects have largely emerged from pre-clinical studies, including modulation of hepatic de novo lipogenesis, fatty acid β-oxidation, oxidative stress, inflammation, and insulin resistance pathways [5,6]. Nevertheless, the overall evidence on this contribution remained characterized by substantial heterogeneity in study design, subject characteristics, dosage, intervention duration, and outcome assessment. In all, this work will contribute to guiding future research in bioactive compounds and underscores the need for standardized methodologies and adequately powered clinical trials to establish their true therapeutic value in MASLD.

Beyond the most classically studied bioactive compounds described in the scoping review, nutritional research emerges describing unexplored food-plant extracts to tackle MASLD. Examples of these emerging approaches are presented in pre-clinical murine studies by Riilo et al. (Contribution 2) and by Besné-Eseverri et al. (Contribution 3), where novel polyphenols extracts have been evaluated.

The study of Riilo et al. (Contribution 2) uses a polyphenolic fraction from Bergamot (*Citrus bergamia*), a Citrus family plant native to Southern Italy, to prevent hepatic lipid accumulation in a Cafeteria diet mouse model. This study described that Bergamot polyphenolic fraction attenuated the hepatic lipogenesis trough reduced ATP citrate lyase (Acly) gene and protein expression levels. In parallel, the authors showed that the Bergamot polyphenolic fraction exerted restoring effect on the autophagic function (evidenced by the increase in LC3-II protein levels), and on short-chain fatty acid (SCFA) production, (especially acetic, butyric, and propionic acids), both mechanisms also associated with mitigation of hepatocellular injury.

The study by Besné-Eseverri et al. (Contribution 3) assessed the impact of phenolic extracts from *Opuntia ficus-indica* and *Opuntia stricta* var. *dillenii*, both members of the Cactaceae family. In the study, different doses of the polyphenol extract were evaluated in a high-fat, high-fructose diet which induced MASLD in Wistar rats. Amongst the four treatments, considering the low and high doses of both extracts, the lower dose of the *Opuntia ficus-indica* phenolic extract was the most effective, with a partial prevention of liver steatosis through readouts such as triglyceride accumulation and histological damage. Mechanistically, phenolic extracts from *Opuntia ficus-indica* exerted their effects by reducing acetyl-CoA carboxylase (ACC) activity—the first committed step in fatty acid synthesis—and by downregulating hepatic FATP2 protein expression involved in fatty acid uptake.

Apart from phenolic compounds, there are others bioactive compounds from diet exhibiting a preventative role against MASLD pathophysiology. Coenzyme Q10 (CoQ10) is a lipid-soluble quinone that acts as an electron carrier in the mitochondrial electron-transport chain, helping generate ATP in the inner mitochondrial membrane. CoQ10 supplementation has been used in different studies to treat diseases that have oxidative stress involvement and excessive reactive oxygen species (ROS) production, such as cardiovascular disease (CVD), dyslipidemia, or endothelial dysfunction [7]. However, its effect on MASLD prevention is less explored. The scientific article by Go et al. (Contribution 4) provides new insights into Coenzyme Q10 supplementation as a potential intervention targeting membrane lipid dysregulation in MASH. In this study, the supplementation of mice with Coenzyme Q10 attenuated the histological severity and serum transaminases levels induced by 4 weeks of MCD diet treatment. Mechanistically, the authors have described that Coenzyme Q10 exerted its protective effects of remodeling hepatic phospholipid composition, increasing phosphatidylcholine (PC) species, and reducing phosphatidylethanolamine (PE) species, resulting in an increased hepatic PC-to-PE ratio.

## 3. Functional Foods Within Dietary Patterns

Moving beyond isolated compounds, the role of functional foods is exemplified by the systematic review on extra virgin olive oil (EVOO) by Bernardino et al. (Contribution 5). This contribution synthesized evidence from 25 human studies, 12 of which assessed olive oil alone and 13 evaluated the MD emphasizing extra virgin olive oil. This narrative review presented that EVOO consumption is associated with improvements in hepatic steatosis, liver enzymes, inflammatory markers, and insulin resistance. Overall, the observations from this review suggested that both the quality and quantity of olive oil are important factors affecting its efficacy in the dietary management of MASLD, with EVOO standing out due to its high phenolic content. The authors of this study also highlight that a greater effect is achieved when EVOO is consumed within the context of a Mediterranean dietary pattern, reinforcing the concept that the health effects of individual foods depend on the overall dietary matrix. This work bridges the gap between reductionist and holistic approaches, emphasizing that functional foods could act synergistically within dietary patterns rather than as isolated nutritional components.

## 4. Dietary Patterns as Complementary Approaches to Pharmacological Strategies

Lifestyle modification has long remained the cornerstone of MASLD prevention [4]. However, recent pharmacological advances have included the approval of resmetirom (2024), the first therapy for noncirrhotic MASH with moderate-to-advanced fibrosis [3,4]. Likewise, several other agents remain under investigation, awaiting the results of larger clinical trials. Importantly, tirzepatide (TZP), a dual GIP/GLP-1 receptor agonist which improves glycemic control and reduces bodyweight in patients with obesity and type II diabetes, has been evaluated in a phase II trial involving participants with MASH and moderate or severe fibrosis [8]. In this context, the study by Schiavo et al. (Contribution 6) provided a particularly relevant clinical insight by directly comparing two dietary approaches in patients receiving tirzepatide therapy. The authors evaluated the effects of a low-energy ketogenic diet (LEKD) versus a conventional low-calorie diet (LCD), both combined with TZP pharmacological treatment. Although both interventions led to significant improvements in weight, hepatic steatosis, and metabolic parameters, the ketogenic approach resulted in greater improvements in liver fat and stiffness, as well as metabolic outcomes. These findings suggest that dietary composition remains a critical determinant of therapeutic efficacy, even in the presence of highly effective pharmacological agents.

It is important to note that this study introduces the concept of synergistic interactions between pharmacotherapy and nutrition, highlighting that metabolic benefits may be optimized when specific dietary strategies—such as carbohydrate restriction and nutritional ketosis—are combined with incretin-based treatments. This represents a shift toward integrated therapeutic models in MASLD, where diet is not replaced by pharmacology but rather acts as a complementary and amplifying factor.

## 5. Conclusions and Future Directions

MASLD has gained increasing attention in recent decades due to its high global prevalence. Numerous bioactive compounds and nutritional approaches have been investigated to address this complex metabolic disorder in experimental models and clinical interventions, identifying several underlying metabolic pathways as potential nutritional and therapeutic targets. This Special Issue has gathered six contributions, providing a review of key bioactive compounds and substances employed in primary interventions. It also includes pre-clinical studies providing evidence on novel bioactive compounds and plant extracts, as well as insights into their main mechanisms of action against MASLD. These mechanisms include inhibition of hepatic lipogenesis, remodeling of hepatic lipidome, restoration of autophagic function, and modulation of the gut–liver axis through regulation of the short-chain fatty acid profile.

The contributions also emphasized that the efficacy of functional foods, such as olive oil, depends not only on their quantity and quality but also on their integration within overall healthy dietary patterns. In addition, nutrition remains essential, even in the “era” of advanced pharmacological therapies with which it has potential synergistic effects that warrant further investigation. Finally, the studies included here also underscore several current limitations in the field, particularly the heterogeneity in study design, intervention duration, dosage, and outcome assessment. Future research should therefore prioritize long-term, well-designed randomized clinical trials with standardized and clinically meaningful endpoints, together with the integration of omics technologies and precision nutrition approaches that can facilitate translation into real-world clinical practice.
